# Mitigating Firearm Suicide with Trusted Messengers in Health Care

**DOI:** 10.1017/jme.2025.10154

**Published:** 2025

**Authors:** Michael R. Ulrich, Cassandra Devaney

**Affiliations:** 1 School of Public Health & School of Law, Boston University, United States; 2 Center for Advancing Research, Methods, and Scholarship in Gun Injury Prevention, University of Connecticut, United States

**Keywords:** Firearms, Guns, Suicide, Prevention, Health care

## Abstract

Voluntary firearm safety actions avoid Second Amendment scrutiny, but rely on individuals recognizing their own risks. This could be aided by a network of healthcare professionals that have received proper training and information about all available tools to help prevent firearm-related suicide attempts, and combining the trust of clinicians and firearm owners could represent an opportunity to inform and educate in a manner that will engage patients.

In their article *Psychiatrists’ Awareness and Use of Voluntary Self-Prohibition as a Firearm Suicide Prevention Tool in Virginia*, Barks, Frattaroli, and Nestadt examine psychiatrists’ awareness of Virginia’s law allowing people to add their name to a no-buy list that bars them from purchasing firearms.^1^ Voluntary self-prohibition (VSP) is a suicide prevention tool that relies on individuals recognizing their risk outside of times of suicidal ideation to ensure they are unable to purchase firearms from sellers that participate in background checks.^2^ As the authors note, psychiatrists can play an important role in implementation because of their clinical assessment expertise and their ability to utilize an existing, trusting relationship.^3^ Since this type of law exists in only four states — the first passing in 2019 — the likelihood of psychiatrists raising this option depends on their awareness.^4^ The authors found that over 90% of respondents were unaware of Virginia’s VSP law, though over 80% agreed it is a useful suicide prevention tool.^5^ Though not included in their study, Virginia residents may be similarly unaware of the law.

The near universal lack of psychiatrist awareness for Virginia’s VSP law highlights the need for the dissemination of educational materials and training about voluntary firearms safety policies in conjunction with the law itself. While health care professionals are aware of the high lethality of firearms, they rarely ask patients about firearms and in some circumstances believe they are legally prohibited from doing so.^6^ One study found that nearly half of responding emergency physicians and residents did not feel comfortable asking patients about firearms given their own lack of knowledge.^7^ This demonstrates that training on firearm safety counseling could be beneficial, but knowledge is only one step toward utilization. The same study examining emergency physician comfort discussing firearms with patients also found that only a quarter of emergency physicians agreed that counseling would impact patients’ secure storage behaviors.^8^

The voluntary aspect of VSP laws helps avoid Second Amendment challenges but also limits the potential impact. Suicidal ideation can have quick onset, so proactively preventing the ability to purchase firearms helps minimize risks of impulsive urges that tend to subside in a relatively short amount of time.^9^ Nevertheless, as the number of firearms owners continues to rise, those who may be helped by their inability to purchase a firearm declines.^10^ To be sure, no single policy is capable of addressing the gun violence problem, or even firearm suicide. Laws permitting extreme risk protection orders (ERPOs), for example, are discussed by Barks, Frattaroli, and Nestadt as a complementary policy because they allow firearms already owned to be removed from someone who is at risk of harming themselves or others.^11^ However, these laws, and many others, are being challenged as violating the Second Amendment.^12^ As the Supreme Court continues to ignore empirics on efficacy and impact,^13^ the role of voluntary firearms safety policies may need to take on a larger role.

VSP laws, among other voluntary actions, rely on individuals recognizing their own risks, and this could be aided by a network of healthcare professionals that have received proper training and information about all available tools to help prevent firearm-related suicide attempts. Of course, equally important is that the clinicians are given reason to believe these tools will help. There are safety measures that people can take to reduce the potential for accidental shootings, and those same measures can reduce the risks of suicide attempts with firearms. Secure storage, for example, has been shown to reduce suicide.^14^ An individual who already owns firearms could store their firearm outside of the home, whether it be at a storage facility, shooting range, or with a friend or relative.^15^ Firearms could also be securely stored within the home, and if a key-locked storage device is used, the keys could be given to another trusted person who would be better equipped to hold the key during times of crises. These options similarly require the awareness, understanding, and buy-in of gun owners. Therefore, as with the VSP approach, the impact of these other voluntary policies would almost certainly be buoyed by participation from health care professionals.

Health care workers generally are a trusted source of information.^16^ Research also shows that this trust increases when the patient and clinician share certain characteristics, such as race and gender.^17^ This should be taken into account when considering who may be the best sources to discuss firearms safety options. Another shared characteristic that has been proven useful within the firearms community is gun owners speaking to other gun owners.^18^ Indeed, firearm owners are perhaps best positioned to understand both the dangers of guns in the home and best practices for minimizing those risks. As firearm owners continue to show an openness toward, and in many circumstances embrace, safety training and security measures, combining the trust of clinicians and firearm owners could represent an opportunity to inform and educate in a manner that will engage patients.

However, initial buy-in from clinicians must come first, and characteristics such as race, gender, and firearm ownership have been shown to influence perceptions of the dangers posed by firearms,^19^ including among physicians.^20^ Integrating health care workers in public health policies has a sordid history in this country, where even under the auspices of helping those in need it has resulted in disparate targeting and criminalization of people of color.^21^ While efforts to reduce the risk of harm from guns should be considered a positive, it is important to be mindful that firearm owners are not maligned and ostracized. An inappropriate approach could exacerbate health problems through internalized stigma and reduced interactions with health care professionals, which would be especially problematic for those at risk of suicide. This could underscore the need to for physicians to have standardized questions and information about firearm ownership and safety risks.

While individual discussions between clinicians and patients about firearm safety measures such as VSP laws have a role to play, relying on individual behavior change through educational efforts has historically had the smallest impact among public health interventions.^22^ Nevertheless, they can contribute as part of a more holistic effort to shift cultural norms around firearms and useful safety measures. The gun debate is often framed through opposing ends of the spectrum, but most people believe the Second Amendment protects an individual right, and most people support gun safety measures. Firearm owners are not a monolith and efforts to reduce firearm suicide, and gun violence more broadly, would be better served to keep this in mind. People frequently assert self-defense as their primary justification for ownership,^23^ yet evidence suggests they are not so sanguine when it comes to their fellow firearm owners.^24^ The culture and discussions around firearms must evolve and VSP laws may very well aid in that development. But it is firearm owners, both inside and outside of the health care system, that may be best equipped to have the most significant impact in finding the appropriate balance between protecting rights and safety.
